# Reducing Glutamate Uptake in Rat Hippocampal Slices Enhances Astrocytic Membrane Depolarization While Down-Regulating CA3–CA1 Synaptic Response

**DOI:** 10.3389/fnsyn.2020.00037

**Published:** 2020-08-18

**Authors:** Ipsit Srivastava, Erika Vazquez-Juarez, Maria Lindskog

**Affiliations:** Division of Neurogeriatrics, Center for Alzheimer Research, Department of Neurobiology, Care Sciences and Society, Karolinska Institutet, Solna, Sweden

**Keywords:** EAAT-1, EAAT-2, GLAST, GLT-1, synapse, currents, adaptation

## Abstract

The majority of synaptic activity in the brain consists of glutamatergic transmission, and there are numerous mechanisms, both intra- and inter-cellular that regulate this excitatory synaptic activity. Importantly, uptake of glutamate plays an important role and a reduced level of astrocytic glutamate transporters affect the normally balanced neurotransmission and is observed in many mental disorders. However, reduced glutamate uptake affects many different synaptic mechanisms in the astrocyte as well as in the neuron, and the effects are challenging to delineate. Combining electrophysiological recordings from neurons and astrocytes as well as extracellular glutamate recordings in rat hippocampal slices, we confirmed previous work showing that synaptic stimulation induces a long-lasting depolarization of the astrocytic membrane that is dependent on inward-rectifier potassium channels. We further showed that when glutamate transporters are blocked, this astrocytic depolarization is greatly enhanced although synaptic responses are reduced. We propose that increasing the levels of synaptic glutamate through blocking glutamate transporters reduces the AMPA-mediated synaptic response while the NMDA receptor current increases, contributing to a rise in extracellular K^+^ leading to enhanced astrocytic depolarization.

## Introduction

Glutamate uptake is a key component in the regulation of excitatory synaptic strength. In the central nervous system the astrocytic excitatory amino acid transporters 1 and 2 (EAAT-1 and -2) are responsible for the majority of glutamate uptake (Bergles and Jahr, 1998), and the neuronal transporters EAAT 3–5 contribute to a lesser extent (Rose et al., 2018). Glutamate transporters are crucial for proper neuronal functioning (Anderson and Swanson, 2000; Rose et al., 2017). Interestingly, a reduction in the expression of these transporters is seen in neurodegenerative as well as psychiatric disorders (Gomez-Galan et al., 2013, 2016; Spangaro et al., 2014; Chung et al., 2015; Garcia-Esparcia et al., 2018) and glutamate transporters have been suggested as targets for new pharmacological treatments of mental disorders (Jensen et al., 2015). A reduction in glutamate uptake leads to increased extracellular glutamate and activation of extrasynaptic NMDA receptors as well as presynaptic metabotropic glutamate receptors (Potier et al., 2010), increasing postsynaptic excitation (Tong and Jahr, 1994; Arnth-Jensen et al., 2002) and eventually to excitotoxicity. However, with increased knowledge about the very tight synapse-astrocyte interaction, it is becoming clear that a pathological increase in glutamate affects the neuronal activity in more complex ways than by inducing excessive increase in activity and excitotoxicity (Goncalves-Ribeiro et al., 2019).

In addition to glutamate uptake, astrocytes regulate synaptic activity through release of gliotransmitters (Araque et al., 2014), potassium buffering (Orkand, 1980; Sibille et al., 2014), regulation of extracellular fluid circulation (Iliff et al., 2012) etc. Vice-versa, the astrocytes themselves can be regulated by a plethora of mechanisms, including pressure (Bowman et al., 1992), metabolism (Bélanger et al., 2011), inflammatory signals (Habbas et al., 2015) and, most importantly for their integral role in neural circuits, by neuronal activity (Martin et al., 2015; Adamsky et al., 2018; Covelo and Araque, 2018). Indeed, the close functional and anatomical interaction between perisynaptic astrocytic processes and the synaptic structure, often referred to as the tripartite synapse, is fundamental for a balanced network activity (Araque et al., 1999; Santello et al., 2019).

A prerequisite for the close astrocyte-synapse interaction is that astrocytes can sense neuronal activities. Several mechanisms have been proposed, including metabotropic glutamate receptors (Panatier et al., 2011), metabolic signals (Choi et al., 2012), and extracellular potassium concentration (Bellot-Saez et al., 2017). A considerable attention has been given to intracellular Ca^2+^ signal in astrocytes (Khakh and McCarthy, 2015; Bazargani and Attwell, 2016) whereas much less is known about a change in membrane potential could act as a signal transduction mechanism. Compared to neurons, astrocytes are electrically passive and do not seem to express voltage sensitive channels to any large extent (Steinhauser et al., 1992; Verkhratsky and Nedergaard, 2018). Their low resting membrane potential (approximately −80 mV) and low input resistance are largely determined by the high number of potassium channels (Ransom and Goldring, 1973; Djukic et al., 2007; Dallerac et al., 2013; Hwang et al., 2014). Although astrocytes have been shown to depolarize in response to synaptic response (Zhang et al., 2003; Pannasch et al., 2012) as well as to direct application of glutamate and GABA (Kettenmann and Schachner, 1985) we still lack an understanding of the electric response of astrocytes in normal or pathological conditions.

In this work, we examine the effect of astrocyte membrane potential, mimicking the pathological state of reduced glutamate uptake where the levels of extrasynaptic glutamate is increased by blocking glutamate transporters. We confirm that evoked synaptic activity in the Schaffer collaterals, axons projecting from the CA3 area to the CA1 area of the hippocampus, leads to a distinct, slow depolarization mediated by an increase in extracellular K^+^, and that this depolarization is significantly increased in conditions of increased extracellular glutamate. Interestingly, this response is not a linear readout of the synaptic signal, since an inhibition of glutamate transporters leads to a decrease in the synaptic response concomitant to the increase in astrocytic membrane depolarization. Instead the slow astrocytic membrane depolarization is dependent on an increase in NMDA receptor activation.

## Materials and Methods

### Animals and Husbandry

All experiments were performed using male Sprague Dawley rats (Charles River Laboratories) aged 21–29 days for patch clamp recordings or 6–8 weeks for field recordings. The animals were housed at the Karolinska Institutet animal facility at 12:12 light/dark cycle and kept and used according to local and national regulation. Experiments were performed with the ethical permit granted by the Animal Ethics Committee at the County Administrative Board (Norra Stockholms Djurförsöksetiska Nämnd, approval N13/15).

### Slice Preparation

Rats were deeply anesthetized with isoflurane, decapitated and the brain was quickly removed. For field recordings the brain was placed in ice-cold standard artificial CSF (aCSF) containing in mM: 130 NaCl, 3.5 KCl, 1.25 NaH2PO4, 24 NaHCO3, 10 glucose, 2 CaCl2, and 1 MgCl2 (pH 7.3–7.4, 310–330 mOsm), bubbled with carbogen gas (5% CO_2_, 95% O_2_). Hippocampal horizontal slices (400 μm thick) were prepared using a Leica VT1200S vibratome (Leica Microsystems). Immediately after slicing, sections were transferred into an interphase incubation chamber filled with standard aCSF (in mM: 130 NaCl, 3.5 KCl, 1.25 NaH2PO4, 24 NaHCO3, 10 glucose, 2 CaCl2, and 1.3 MgCl2). The chamber was held at 34°C during the slicing and was subsequently allowed to cool down at room temperature. For patch clamp recordings 300 μm slices were prepared in the same way, with dissection solution containing (in mM): 250 sucrose, 2.5 KCl, 1.4 NaH_2_PO_4_, 26 NaHCO_3_, 10 glucose, 1 CaCl_2_, and 4 MgCl_2_ (310–330 mOsm). The recovery and recording was done in the same standard aCSF solution as above.

### Patch-Clamp Recordings

**FIGURE 1:**
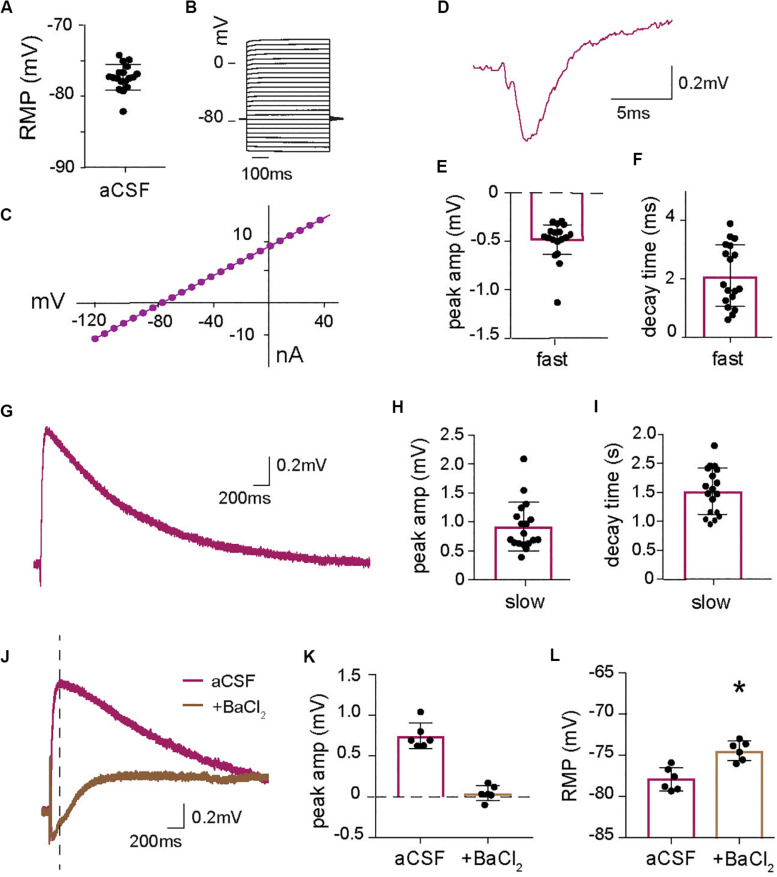
Astrocytic long lasting depolarization induced by increase in extracellular K^+^ at synaptic activity. **(A)** Baseline resting membrane potential (RMP) of all astrocytes recorded in current clamp for this paper (*n* = 18). **(B)** Voltage steps protocol and **(C)** I–V curve of a representative astrocyte showing a linear relationship between current and voltage confirming astrocyte identity. **(D)** Representative trace of the fast astrocytic response to synaptic stimulation with **(E)** mean peak amplitude and **(F)** mean decay time (*n* = 18, including cells also analyzed in Figures 3, 5). **(G)** Representative trace of the long-lasting astrocytic depolarization in response to synaptic activity and quantification of **(H)** peak amplitude and **(I)** decay time (*n* = 18). **(J)** Representative trace of the long-lasting astrocytic depolarization in the absence and presence of barium chloride, BaCl_2_ (100 μM), purple and brown, respectively, with the baseline adjusted. BaCl_2_ application significantly reduces the **(K)** peak amplitude (time of measurement indicated by line in **J**) of the astrocytic long-lasting depolarization (*p* < 0.01, *n* = 6). **(L)** BaCl_2_ application depolarizes the astrocyte resting membrane potential (RMP) (*p* < 0.01, *n* = 6). Electrical stimulation artifact removed from displayed traces for clarity. *Denotes statistical significance.

After a recovery period of at least 1 h, slices were transferred to a submerged recording chamber with perfusion rate 2–3 ml per min with aCSF at 32 ± 1°C, bubbled with carbogen. Picrotoxin (50 μM, Tocris Biosciences) was added to the recording solution to omit effects of inhibitory input. Ag/AgCl electrode was used with borosilicate glass pipettes with a tip resistance of 5–7 MOhm for patching astrocytes, and 4–5 MOhm for neurons. The glass pipettes were filled with a solution containing (in mM): 110 K-gluconate, 10 KCl, 4 Mg-ATP, 10 Na_2_-phosphocreatine, 0.3 Na-GTP, 10 4-(2-hydroxyethyl)piperzine-1-ethanesulfonic acid (HEPES) and 0.2 ethylene glycol tetraacetic acid (EGTA) (pH 7.2–7.4; 270–290 mOsm). Neurons were identified by shape and localization in the pyramidal cell layer, astrocytes were identified by shape and localization in the stratum radiatum. Astrocyte identity was confirmed by a hyperpolarized membrane potential (−75 to −85 mV) and no voltage dependent currents (see Figures 1A–C). Voltage response to an injected current pulse was monitored to ensure the quality of the recording throughout the experiment and data was included only for stable values (<20% variation). Synaptic responses were evoked by electrical stimulation of the Schaffer collaterals (SC) using a bipolar concentric electrode and stimulation intensity to evoke 50–60% of the maximal response was used. A single stimulation pulse was given and the postsynaptic response was averaged over 5 sweeps. Data acquisition was done using Multiclamp 700B amplifier and Clampex 10.0 (Molecular Devices), digitized with Digidata 1440A (Molecular Devices). Glutamate transporter currents were recorded in astrocytes voltage clamped at −80 mV in the presence of NBQX (10 μM) and DL-APV (50 μM) in response to increasing Schaffer collateral stimulation using a bipolar electrode. For neuronal recordings, all neurons were voltage clamped at −65 mV. Traces were analyzed in the pClamp or Minianalysis software (Synaptosoft, United States).

### Field Recordings

After a recovery period of 2 h, slices were transferred to a submerged recording chamber with perfusion rate of 2–3 ml per min with standard aCSF at 32 ± 1°C, bubbled with carbogen gas. Field excitatory postsynaptic potentials (fEPSPs) were evoked at 0.1 Hz by electrical stimulation of the Schaffer collaterals (SC) using a bipolar concentric electrode coupled to an isolated stimulator. Stimulation intensity to evoke 50–60% of the maximal response were used. Responses were recorded in the CA1 using an Ag/AgCl electrode coupled to an amplifier (EXT-02, npi), digitized (Digidata, Molecular Devices, United States) and monitored with the pClamp software (Molecular devices). Traces were analyzed in the pClamp software. A 20 min stable fEPSP baseline was recorded before the slices were perfused with specific drugs (see below). The size of the responses was measured by determining the slope of the linear rising phase of the fEPSP (between 10 and 80% before reaching the peak amplitude). Experiments were normalized to their individual baseline periods. The effect of each specific drug was determined by comparing a 5 min average of the normalized fEPSP slope after treatment (minutes 16–20) to the averaged fEPSP slope of baseline recording (30 sweeps, respectively).

### Glutamate Recordings

R1 ceramic-based microelectrode arrays (MEAs; The Center for Microelectrode Technology, University of Kentucky) containing four vertical-aligned recording sites were used to monitor glutamate concentration in hippocampal slices. The glutamate-sensitive site of the MEA, was coated with glutamate oxidase (0.1 unit/μL; G4001-01, US Biological, United States), BSA (0.8%), and glutaraldehyde (0.1%). An adjacent site, used as a sentinel detector for background noise and non-specific signals, was only coated with BSA and glutaraldehyde. Coated MEAs were allowed to dry for 48 h at room temperature and then electroplated with a size-exclusion *m*-phenylene diamine layer. Prior to every experiment, MEAs were calibrated using a potential of 0.7 V versus an Ag/AgCl reference electrode. Briefly, calibration was performed in PBS solution (0.05 M, 40 ml, pH 7.4, 37°C). After stabilization of the baseline signal, Ascorbic Acid (250 μM), L-Glutamate (3 μM × 20 μM), Dopamine (2 μM), and H_2_O_2_ (8.8 μM) were sequentially added to the calibration beaker. Amperometric signals were acquired at 10 Hz using a FAST-16 mkIV electrochemical recording system (Quanteon LLC, United States) and analyzed off-line. The final concentration of glutamate in the slice was calculated by subtracting the signal recorded by the sentinel to that of the glutamate-sensitive site.

### Drugs

The following (concentrations of) drugs were delivered through the recording aCSF solution: GABA_A_ receptor antagonist: Picrotoxin, PTX (50 μM), AMPA receptor antagonist NBQX (1, 2,3,4-Tetrahydro-6-nitro-2,3-dioxo-benzo[f]quinoxaline-7-sulf- onamide hydrate; 10 μM), NMDA receptor antagonist DL-AP5 (DL-2-Amino-5-phosphonopentanoic acid; 25 μM), EAAT blocker DL-TBOA (DL-threo-beta-Hydroxyaspartic acid; 50 μM), Group II mGluR antagonist LY341495 were from Tocris Bioscience (United Kingdom). Barium chloride, BaCl_2_ (100 μM) and NMDA antagonist MK-801 [(5S,10R)-(+)-5-Methyl-10,11-dihydro-5H-dibenzo[a,d]cyclohepten-5,10-imine; 10 μM] were from Sigma-Aldrich (United States). Both DL-AP5 and MK-801 completely blocks NMDA receptors under these conditions and are considered equivalent.

### Statistics

Unless otherwise noted, data is presented as average value ± SEM. Statistical analysis was done using the GraphPad software and assuming normal distribution. Statistical significance is analyzed with Student *t*-test when two groups are compared or with ANOVA followed by Tukey’s multiple comparison for comparing values before and after treatment in the same cell or Bonferroni’s multiple comparison to compare unpaired values from different cells or slices.

## Results

### Synaptic Activation Generates a Slow Depolarization of Astrocytes

Patch-clamp recordings from astrocytes in the stratum radiatum revealed an average resting membrane potential (RMP) of −77.33 ± 0.43 mV (*n* = 18; Figure 1A). The cells did not respond with action potentials at depolarizing voltage pulses up to +40 mV (Figure 1B) and showed a linear I–V curve (Figure 1C). Synaptic activity triggered by stimulation of Schaffer collaterals generated a fast membrane potential response (Figure 1D) with an average peak amplitude of 0.49 ± 0.05 mV (Figure 1E) and decay time of 2.11 ± 0.25 ms (*n* = 18; Figure 1F). This fast response has previously been described and is a reflection of the field EPSP that can be measured in the astrocyte due to the low membrane resistance (Henneberger and Rusakov, 2012) and is present in all our astrocytic recordings when the Schaffer collateral is stimulated. In addition, we identified a long-lasting depolarization of the astrocytic membrane in response to collateral stimulation (Figure 1G) with a decay time of 1.51 ± 0.93 s and an average peak amplitude of 0.92 ± 0.10 mV (*n* = 18; Figures 1H,I). Since astrocytic membrane potential is largely set by potassium, we examined the role of K^+^ channels in this response by blocking them with 100 μM BaCl_2_. As expected, BaCl_2_ resulted in a significantly depolarized astrocytic resting membrane potential compared to baseline (−74.36 ± 0.48 mV with BaCl_2_ versus −77.83 ± 0.57 mV before, *p* < 0.01, *n* = 6; Figure 1L). To investigate the effect of blocking of potassium channels on the slow depolarization evoked by synaptic activity the change in potential compared to the baseline was measured and showed that BaCl_2_ completely blocked the depolarization (0.04 ± 0.04 mV, compared to 0.74 ± 0.06 mV in control; *p* < 0.01, *n* = 6; Figures 1J,K). These results suggest that astrocytes respond to synaptic activity with a slow depolarization mediated by an increase in extracellular K^+^ triggered by synaptic activity. The recordings display a biphasic response to BaCl_2_, with a slow depolarization appearing about 0.5 s after stimulation. Although we did not explore this effect, it indicates that blocking K^+^ channels affects many cellular processes, including the Na^+^/K^+^-ATPase, that can affect the membrane potential.

### Astrocytic Response to Glutamate Transporter Blocking

**FIGURE 2:**
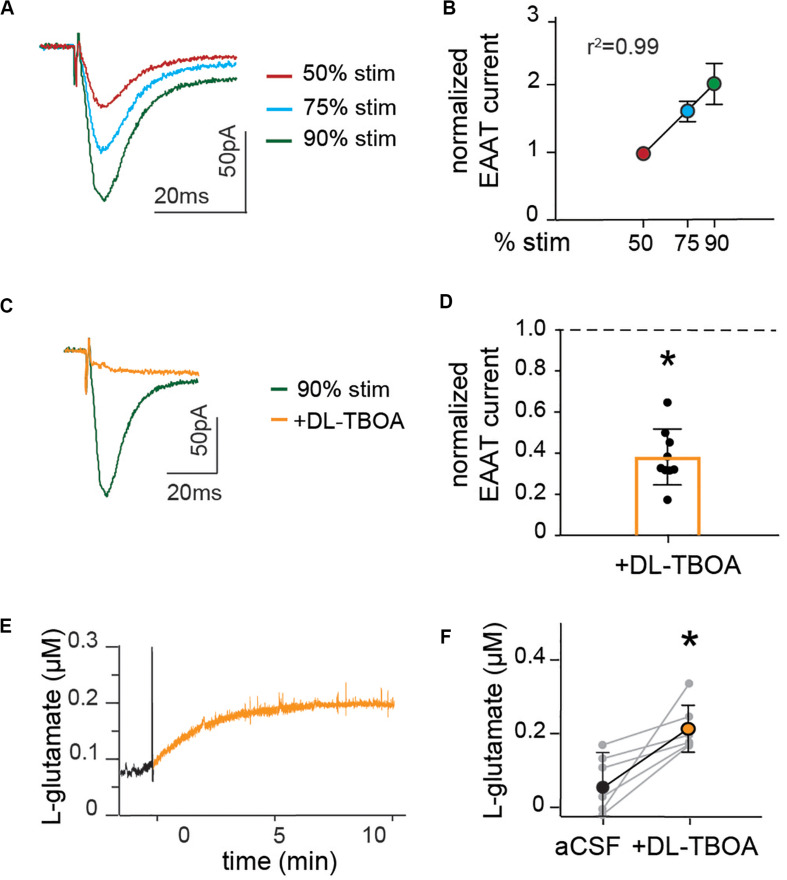
Astrocytic glutamate transporter currents increase linearly with synaptic stimulation and blocking glutamate transporters doubles baseline extracellular levels of glutamate. **(A)** Representative trace of EAAT current measured in the presence of NBQX and AP5, at schaffer collateral stimulation corresponding to 50% (red), 75% (blue), and 90% (green) of maximal stimulation strength. **(B)** Peak amplitude of EAAT current normalized to the amplitude at 50%, showing that glutamate transporter currents increase linearly with increase in stimulation strength. **(C)** Representative recordings of EAAT current in the presence of EAAT blocker DL-TBOA (50 μM). **(D)** Average glutamate transporter currents normalized to current before the application of EAAT blocker are significantly reduced in the presence of DL-TBOA (*p* < 0.01, *n* = 9). Enzyme coated microelectrode were used for recording of glutamate concentration. **(E)** Representative figure showing L-glutamate recordings under baseline conditions (black) and after application of DL-TBOA (50 μM; orange). **(F)** Glutamate concentrations before and 15 min after DL-TBOA (50 μM) application shown for each slice, the averages are significantly different (*p* < 0.05, *n* = 6). Electrical stimulation artifact removed from displayed traces for clarity. *Denotes statistical significance.

Glutamate transporters are critical for regulating synaptic strength, and they are highly relevant target for the treatment of many mental disorder. When glutamate is released through presynaptic stimulation of Schaffer collaterals, a glutamate transporter mediated inward current that can be recorded in patch-clamped astrocytes in the presence of AMPA and NMDA receptor inhibitors NBQX (10 μM) and AP5 (25 μM). In fEPSP recordings, with synaptic release triggered by Schaffer collateral stimulation, we identified the stimulation triggering 50, 75, and 90% of maximal fEPSP response. This stimulation generated a linear increase of glutamate transporter currents (*r*^2^ = 0.99, slope = 0.03, *n* = 10; Figures 2A,B), suggesting that under basal conditions, the main determinant of glutamate transporter activity is extracellular glutamate concentrations. The current’s identity was confirmed by applying the glutamate transporter blocker DL-TBOA (50 μM) that reduced the fast component of the current (peak amplitude 0.38 ± 0.04 of control, *p* < 0.01, *n* = 9; Figures 2C,D), leaving a slow residual current previously described as potassium mediated (Cheung et al., 2015).

We further assessed the effect of glutamate transporter blocking on the levels of ambient glutamate. Extracellular glutamate concentrations were recorded in slices using an enzyme-coated microelectrode coupled to amperometric detection and DL-TBOA was applied to the slice to block astrocytic as well as neuronal glutamate transporters. The amount of glutamate measured increased gradually after DL-TBOA bath perfusion (Figure 2E), reaching a significant twofold increase from 61 ± 4 to 220 ± 3 nM (*p* < 0.05, *n* = 6, Figure 2F) 15 min after application of DL-TBOA.

### Astrocytic and Neuronal Response to Acute Blocking of Glutamate Transporters

**FIGURE 3:**
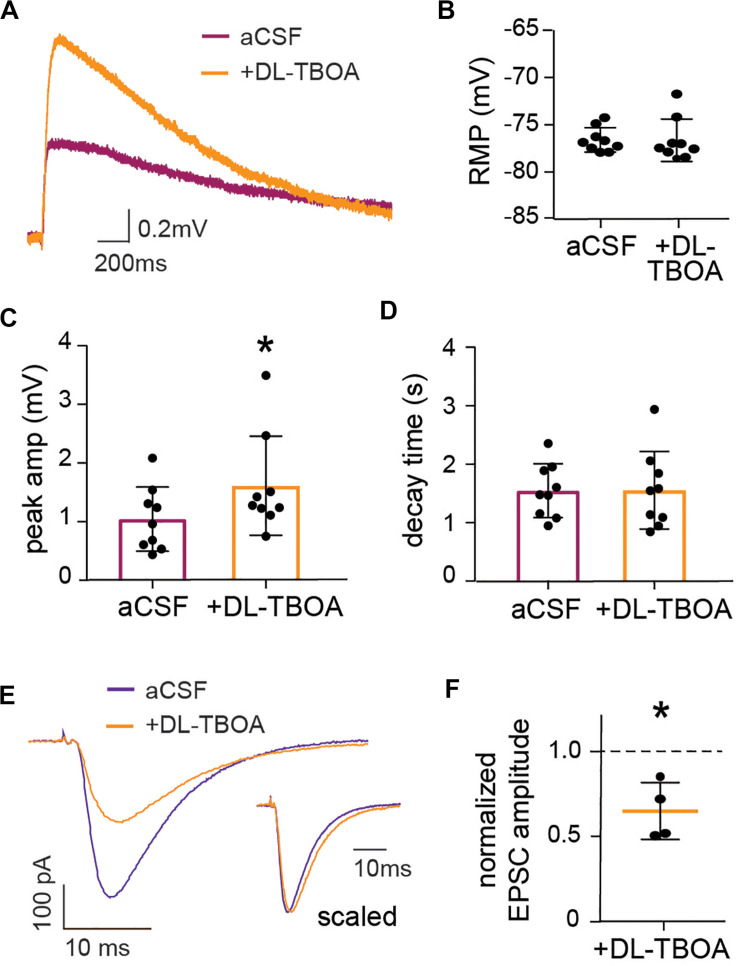
EAAT blocking enhances astrocytic membrane depolarization while reducing neuronal evoked EPSC. **(A)** Representative trace showing astrocytic long-lasting depolarization under control conditions (purple) and 2 min after DL-TBOA application (orange). **(C)** DL-TBOA significantly increases the peak amplitude of the long-lasting depolarization (*p* < 0.01, *n* = 9, where 6 out of 9 cells are the same as in Figure 5) with no significant changes in the **(D)** decay time and **(B)** resting membrane potential (RMP). Voltage-clamp recordings from individual neurons held at −65 mV during stimulation of Schaffer collateral under control conditions and in the presence of glutamate transporter blocker DL-TBOA. **(E)** Representative trace of evoked EPSC before (blue) and 2 min after DL-TBOA application (orange), insert shows traces with amplitude scaled. **(F)** Peak amplitude of evoked EPSC normalized to before application of DL-TBOA for each cell, the average amplitude is significantly reduced in presence of DL-TBOA compared to before (*p* < 0.05, *n* = 4). Electrical stimulation artifact removed from displayed traces for clarity. *Denotes statistical significance.

To investigate the response of astrocytes to reduced glutamate uptake, we recorded membrane potential changes in patched astrocytes in response to Schaffer collateral stimulation in the absence or presence of DL-TBOA (50 μM) to block the glutamate transporters. DL-TBOA application significantly increased the average peak amplitude of astrocytic depolarization from 1.04 ± 0.18 mV to 1.61 ± 0.28 mV (*p* < 0.01, *n* = 9; Figures 3A,C) without modifying the decay time (1.55 ± 0.22 s in the presence of DL-TBOA compared to 1.55 ± 0.15 s at baseline, *n* = 9; Figure 3D). DL-TBOA had no significant effect on the RMP (−76.6 ± 0.43 mV before DL-TBOA compared to −76.6 ± 0.75 mV after treatment, *n* = 9; Figure 3B). A possible explanation for the increased astrocytic depolarization after glutamate transporter inactivation is that the increase in extracellular glutamate (Figure 2) leads to increased neuronal activity, thus enhancing the synaptically evoked depolarization. To confirm this, we recorded the evoked EPSC from CA1 neurons in response to Schaffer collateral stimulation. To our surprise, blocking glutamate transporters with DL-TBOA reduced the evoked EPSC peak amplitude significantly to 0.65 ± 0.08 of control (*p* < 0.05, *n* = 4; Figures 3E,F). When the recordings were scaled to the same amplitude (Figure 3E, inset) a slight and not significant increase of the decay time was observed, from 12.72 ± 1.5 ms at baseline to 14.6 ± 1.0 ms in the presence of DL-TBOA (*p* = 0.14, *n* = 4).

### Blocking Glutamate Transporters Induces a NMDA Mediated Reduction of EPSP Amplitude

**FIGURE 4:**
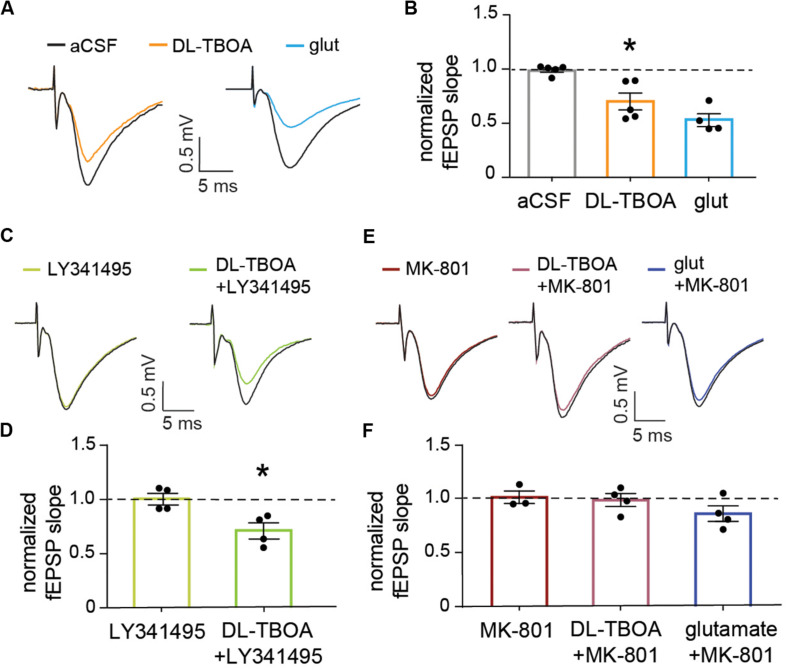
EAAT blocking or exogenous glutamate reduce the evoked synaptic population response in an NMDA receptor-dependent manner. **(A)** Representative traces showing baseline field EPSPs evoked by electrical stimulation of Schaffer collateral and recorded in the striatum radiatum of the CA1 (black traces) and 15 min after 50 μM DL-TBOA or 1 mM glutamate application (orange and blue traces, respectively). **(B)** The fEPSP reduction induced by DL-TBOA and 1 mM glutamate was significantly different when compared to control experiments measured at the same time point after a mock change of perfusion solution (*p* < 0.05, *n* = 5 for DL-TBOA and *p* < 0.01, *n* = 5 for 1 mM glutamate). **(C)** Representative traces showing baseline fEPSP responses (black traces) and 15 min after 10 μM LY341495 alone or combined with DL-TBOA. **(D)** Inhibition of mGlu receptors with LY341495 did not modify either the fEPSP response (*n* = 4) or the significant reduction in amplitude induced by DL-TBOA (*n* = 4). **(E)** Representative traces showing fEPSP responses at baseline (black traces) and 15 min after 10 μM MK-801 alone or combined with 50 μM DL-TBOA or 1 mM glutamate. **(F)** Blocking NMDA receptors fully prevent the reduction in the fEPSP response induced by 50 μM DL-TBOA (*n* = 4) or 1 mM glutamate (*n* = 4). *Denotes statistical significance.

To understand the effect of glutamate transporter blocking on the overall synaptic activity, we turned to extracellular recordings of evoked field EPSPs (fEPSP) at Schaffer collateral-CA1 pyramidal cell synapses. Again, DL-TBOA (50 μM) treatment reduced the normalized fEPSP to 0.67 ± 0.04 (*n* = 5; Figures 4A,B) of baseline as measured by the slope of the response. This decrease was significantly different from control slices in which the normalized fEPSP remained at 0.98 ± 0.03 (*p* < 0.05; *n* = 5) of baseline at the same timepoint after mock change of perfusion solution. Adding glutamate (1 mM) to the perfused aCSF induced a similar reduction in fEPSP response as DL-TBOA (0.53 ± 0.06 of baseline, *p* < 0.01, *n* = 4; Figures 4A,B), confirming that DL-TBOA indeed mediated the synaptic adaptation through elevating the levels of extracellular glutamate. LY341495 to inhibit presynaptic metabotropic glutamate receptors and MK-801 to block NMDA receptors were then used to understand how the decreased synaptic response was mediated. In the presence of 200 nM LY341495 (Losonczy et al., 2003) the evoked fEPSP was still significantly decreased after DL-TBOA treatment (0.72 ± 0.07 of baseline, *p* < 0.05, *n* = 4; Figures 4C,D), however, in the presence of MK-801 (10 μM) the recorded response remained unaltered from baseline level both in the presence of DL-TBOA (0.98 ± 0.05, *n* = 4) and 1 mM glutamate (0.86 ± 0.07, *n* = 4, Figures 4E,F). MK-801 alone did not change the fEPSP slope (1.01 ± 0.06, *n* = 3). This result suggests that the observed reduction in synaptic strength after blocking glutamate transporters is the result of an active homeostatic response to increased extracellular levels of glutamate orchestrated by the NMDA receptor activation and is in line with previous work showing that glutamate overload can induce an NMDA receptor mediated AMPA receptor removal from the synapse (Siddoway et al., 2014).

### A Switch in AMPA/NMDA Activity After Glutamate Transporter Blocking

**FIGURE 5:**
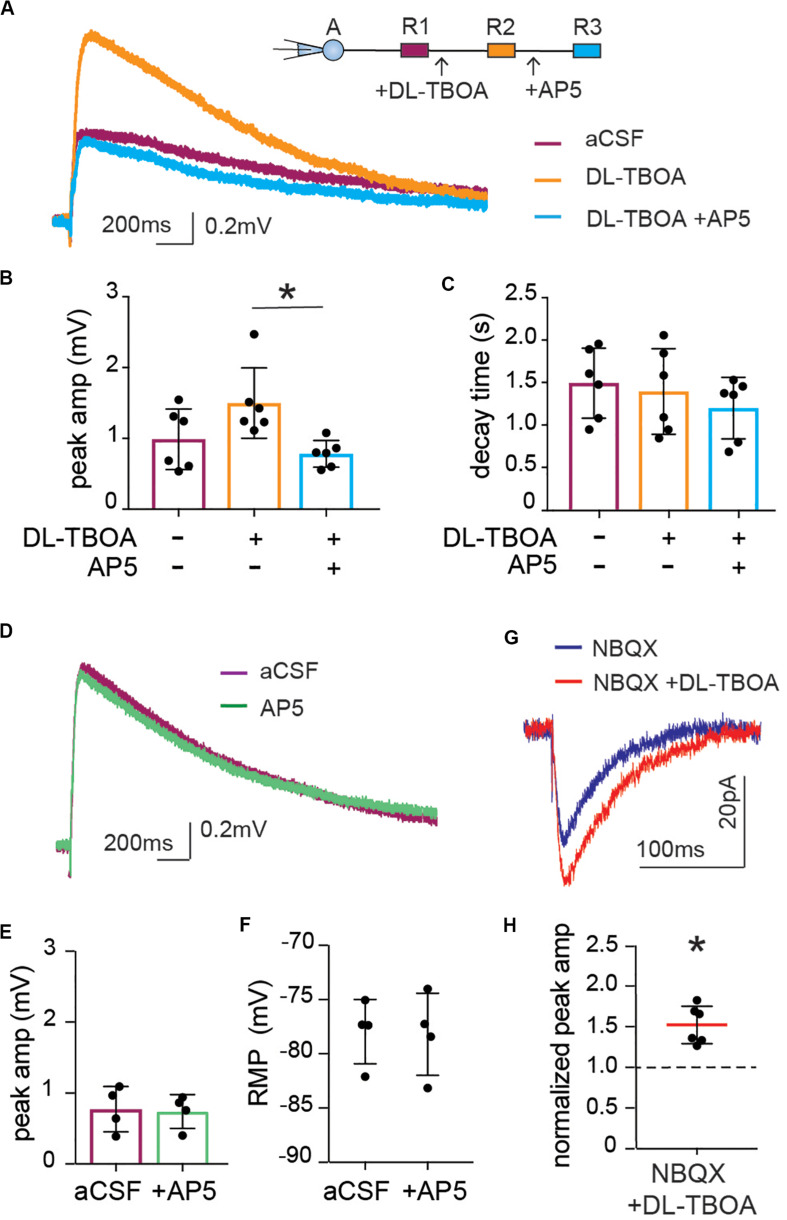
Enhanced astrocytic depolarization after glutamate transporter blocking is dependent on increase in NMDA receptor activity. **(A)** Representative trace of astrocytic depolarization at baseline (purple), after DL-TBOA application (orange) and in the presence of DL-TBOA as well as NMDA receptor inhibitor AP5 (blue). **(B)** The significant increase in peak amplitude of astrocytic depolarization in the presence of DL-TBOA (50 μM) was completely inhibited when AP5 (25 μM) was added along with DL-TBOA (*n* = 6), **(C)** with no difference in decay time in any of the three conditions. **(D)** Representative trace showing the synaptically induced astrocytic depolarization before and after application of AP5 (25 μM, green). **(E)** AP5 does not significantly change peak amplitude or **(F)** resting membrane potential (*n* = 4). Neuronal NMDA responses were recorded as evoked EPSC in patch-clamped neurons by stimulation of Schaffer collateral in the presence of the AMPA receptor antagonist NBQX. **(G)** Representative trace of NMDA component of EPSC recorded before (blue) and after DL-TBOA application (red). **(H)** DL-TBOA significantly increased the average peak amplitude of the synaptically evoked NMDA current (*p* < 0.01; *n* = 6). Electrical stimulation artifact removed from displayed traces for clarity. *Denotes statistical significance.

The fact that astrocytic depolarization is increased, whereas synaptic activity is reduced to less than half when glutamate transporters are blocked, shows that the slow astrocytic membrane depolarization is not a linear readout of synaptic potentials. However, NMDA receptors, as well as AMPA receptors are permeable to K^+^, and an increase in NMDA receptor activation results in an increase of extracellular K^+^ (Shih et al., 2013). Indeed, application of the NMDA receptor inhibitor AP5 completely reversed the increase in synaptically evoked membrane depolarization induced by DL-TBOA from 1.50 ± 0.20 mV in the presence of DL-TBOA to 0.78 ± 0.07 mV when AP5 was added to the slice (*p* < 0.05 compared to DL-TBOA), which was not significantly different from the astrocytic depolarization in response to synaptic activity before the drugs were added; (0.99 ± 0.17 mV, *n* = 6; Figures 5A,B). Again, there was no difference in decay time (control 1.49 ± 0.17 s, DL-TBOA 1.39 ± 0.21 s, DL-TBOA + AP5 1.2 ± 0.15 s, *n* = 6; Figure 5C). Blocking the NMDA receptor alone did not change the astrocytic RMP (control −77.96 ± 1.48 mV, AP5 −78.21 ± 1.89 mV; Figure 5F). Interestingly, AP5 application alone, in the absence of DL-TBOA did not have any effect on the stimulation induced astrocytic membrane depolarization (peak amplitude control 0.77 ± 0.16 mV, in the presence of AP5 0.74 ± 0.12 mV, *n* = 4; Figures 5D,E) suggesting that the NMDA receptors are only activated enough to affect astrocytic membrane potential when glutamate transporters are blocked and extracellular glutamate is increased.

The slight shift in decay time observed in the neuronal EPSC after DL-TBOA application (Figure 3E) suggested that NMDA currents may be increased in this condition, however, this effect was masked by the fast AMPA response. Indeed, by recording the evoked EPSC of pyramidal neurons in the CA1 in presence of AMPA receptor blocker NBQX to isolate the NMDA current, we confirmed that blocking glutamate transporters does indeed lead to an increase in NMDA currents in neurons. In contrast to the effect on the AMPA mediated current (Figures 3E,F), DL-TBOA application significantly increased the peak amplitude of evoked EPSC to 1.52 ± 0.09 of control (*p* < 0.01, *n* = 6; Figures 5G,H).

## Discussion

In this work, we show that astrocytes in the hippocampus depolarize upon synaptic stimulation. The slow time course and the fact that the depolarization can be blocked by BaCl_2_ indicate that the depolarization is mediated by rises in extracellular potassium (Ballanyi et al., 1987; Meeks and Mennerick, 2007), a finding consistent with K^+^ currents recorded under similar conditions (Sibille et al., 2014). Interestingly, the depolarization is not a linear read-out of the synaptic EPSC. When we block glutamate transporters, the astrocytic depolarization is increased, whereas the postsynaptic neuronal response, as recorded either as a single cell EPSC (Figures 3E,F) or a field EPSP (Figures 4A,B), is decreased. We also show that the increased astrocytic depolarization after blocking glutamate transporters is mediated by NMDA receptors. Taken together, our results reinforce the close interaction and finely tuned relationship between neurons and astrocytes at the synapse. We propose that a reduced glutamate uptake by the astrocytes induces a rearrangement of the synapse with a down regulation of synaptic AMPA receptor response but an increase in extrasynaptic NMDA receptor activation, leading to an increase in extracellular potassium that is sensed by the astrocyte through an increased membrane depolarization. This interaction is compatible with previous work, using the method of intrinsic optical signal (Woo et al., 2019).

The importance of astrocytes to buffer extracellular K^+^ is well described (Kofuji and Newman, 2004), but our work shows that K^+^ can also be a signal, in contrast to what have been proposed in an *in silico* model (Savtchenko et al., 2018). Barium sensitive potassium channels are well-known to set the resting membrane potential of astrocytes (Olsen and Sontheimer, 2008). Since potassium currents are the main effectors of the slow depolarization, our data suggests that in conditions of high glutamate (in our case when glutamate transporters are blocked), the activation of presumably extrasynaptic NMDA receptors generates currents that substantially contribute to extracellular potassium levels (Lackington and Orrego, 1986; Shih et al., 2013) and enhances the synaptically induced astrocytic membrane depolarization. An alternative explanation would be that the increased activation of NMDA receptors causes a depolarization of the postsynaptic cell, leading to an increased neuronal firing. However, the time course of the astrocytic depolarization is constant under control and DL-TBOA application, and only the peak amplitude is affected. This makes it unlikely that the effect is due to increased neuronal firing, since this would have led to multiple peaks, or a shift in decay time due to potassium build up after each action potential.

The interdependence of neuronal and astrocytic responses and the fast adaptation of the responses impose particular challenges to study them and to specifically pinpoint the localization of specific mechanisms. Our results do not, for example, allow us to rule out that part of the increase in astrocytic depolarization after DL-TBOA is due to NMDA receptors expressed on the astrocytes. The existence of NMDA receptors on astrocytes is controversial (Dzamba et al., 2013), recordings of NMDA currents in pure astrocytic cell cultures show that astrocytes have the capability to express NMDA receptors (Kettenmann et al., 1984), but whether this is true *in vivo* is less certain and difficult to tease out due to the interdependency of the synaptic and astrocytic events. Recent single-cell sequencing data show that astrocytes express very low levels of NMDA mRNA (Skowronska et al., 2019) and their functional role is unclear. The slow time course and consistent decay-time in our experiments and the fact that neuronal NMDA currents are increased in the presence of DL-TBOA, are compatible with the hypothesis that increased astrocytic membrane potential is mediated through a rise in extracellular K^+^ through increased activation of neuronal NMDA receptors. The possibility that the depolarization is mediated by extracellular K^+^ originating from an increase in action potentials (triggered by NMDA receptor induced depolarization of the postsynaptic neuron), seems less likely based on the constant time course of the depolarization in the absence and presence of DL-TBOA. Our model of explanation is also compatible with previously published work where astrocytic currents were recorded upon NMDA receptor activation (Schipke et al., 2001). On the other hand, work from acutely isolated astrocytes does point to the presence of functional NMDA receptors on astrocytes (Lalo et al., 2006). In our experiments, the NMDA receptor inhibitor AP5 does not have any effect in the absence of DL-TBOA suggesting that extrasynaptic NMDA receptors are involved, activated only when the blockade of glutamate transporters causes an overflow of glutamate beyond the synaptic cleft.

Although astrocytic membrane depolarization is an understudied phenomenon, there is now accumulating evidence that membrane depolarization in astrocytes is functionally relevant. It has been shown that depolarization of cortical astrocytes can increase intracellular Ca^2+^ (Wu et al., 2015) and glutamate transporter currents are affected by changes in astrocyte membrane potential (Tanui et al., 2016; Lebedeva et al., 2018). Interestingly, the fast decrease in the amplitude of the fast neuronal postsynaptic potential in response to high levels of extracellular glutamate can be an adaptation to prevent runaway excitation. In contrast, the astrocytic response to increases in extracellular glutamate, measured as the membrane depolarization, is enhanced. Thus, the signal transduction through astrocytic membrane potential can be especially important at situations with high levels of glutamate and can potentially induce adaptation of the network within a longer timeframe, consistent with the idea that astrocytes are important to sustain a balanced neuronal network activity over time.

## Data Availability Statement

The raw data supporting the conclusions of this article will be made available by the authors, without undue reservation, to any qualified researcher.

## Ethics Statement

The animal study was reviewed and approved by Stockholms Norra Försöksdjursetiska nämnd.

## Author Contributions

IS and ML conceived and designed the work together with EV-J. EV-J and IS performed the experiments. All authors contributed in analysis and interpretation of the data as well as writing the manuscript.

## Conflict of Interest

The authors declare that the research was conducted in the absence of any commercial or financial relationships that could be construed as a potential conflict of interest.
